# Ulcerative Colitis and Its Association with *Salmonella* Species

**DOI:** 10.1155/2016/5854285

**Published:** 2016-01-20

**Authors:** Manish Kumar Tripathi, Chandra Bhan Pratap, Vinod K. Dixit, Tej Bali Singh, Sunit K. Shukla, Ashok K. Jain, Gopal Nath

**Affiliations:** ^1^Department of Gastroenterology, Institute of Medical Sciences, Banaras Hindu University, Varanasi 221005, India; ^2^Department of Microbiology, Institute of Medical Sciences, Banaras Hindu University, Varanasi 221005, India; ^3^Department of Community Medicine, Institute of Medical Sciences, Banaras Hindu University, Varanasi 221005, India

## Abstract

Ulcerative colitis (UC) is characterized by presence of ulcer in colon and bloody diarrhea. The present study explores the possibility of association between* Salmonella* and ulcerative colitis. The present study comprised 59 cases of UC, 28 of colon cancer (CC), 127 of irritable bowel syndrome (IBS), and 190 of healthy control. The serological study was done by Widal and Indirect Haemagglutination Assay (IHA) for ViAb. Nested PCR was performed targeting* fliC*,* staA,* and* stkG* gene for Typhi and Paratyphi A, respectively. A total of 15.3% patients were positive for* Salmonella* “O” antigen among them 18.6% UC, 35.5% CC, 12.6% IBS, and 15.3% healthy control. A total of 36.9% patients were positive for “H” antigen including 39.0%, 57.1%, and 67.7% UC, CC, and IBS, respectively. About 1.73% show positive agglutination for AH antigen including 3.4%, 3.6%, and 1.6%, UC, CC, and IBS. A total of 10.89% were positive for ViAb. While 6.8% of UC, 10.7% of CC, 11.0% of IBS, and 12.1% of healthy subjects were positive for the antibody, the PCR positivity rates for* Salmonella* specific sequences were 79.7% in UC, 53.6% in CC, 66.1% in IBS, and 16.3% in healthy controls. The present study suggested that higher prevalence of* Salmonella *might play important role in etiopathogenesis of UC, IBS, and CC.

## 1. Introduction

Ulcerative colitis (UC) is defined as chronic idiopathic inflammatory condition of gastrointestinal tract characterized by presence of blood in diarrhoeal stool [1]. UC is an inflammatory bowel disease (IBD). The other diseases included under IBD, that is, Crohn's disease (CD) and irritable bowel syndrome (IBS), are also of unknown aetiology [2]. Efforts are being made for last few decades to find out specific pathogen/s causing UC but without any success [3]. The incidence of UC has been observed to be varying with socioeconomic and geographical conditions. While UC is more prevalent in developing tropical countries, prevalence of CD dominates in developed temperate climate countries [4, 5]. Recent trends indicate that in Western Europe and North America (with better hygiene) the figure for incidence of UC is either stabilized or decreasing, and developing countries are now being reported to have very high prevalence of UC [4]. However, CD remains almost an insignificant problem of underdeveloped world. It is very well known that many of the enteric pathogens, that is,* Salmonella *spp.,* Campylobacter *spp.,* Escherichia coli, Shigella *spp.,* Yersinia enterocolitica, Listeria monocytogenes, Mycobacterium *species,* Clostridium difficile*, and so forth, and several of parasitic, viral, and fungal agents are highly prevalent in countries with poor sanitary conditions [6–11]. Further, very recently a study from North India has reported that low hygiene and exposure to infections may be associated with increased risk of UC [12]. The predominance of UC over CD in underdeveloped countries along with the reports showing increased short- and long-term risk of inflammatory bowel diseases after* Salmonella* and* Campylobacter enteritis* infections [9–11] suggests possible role of infectious agents causing UC. Therefore, we can say that a specific pathogen has not been detected yet in IBD cases. However, we can say that failure in detection of such pathogens may be due to inadequacy of methods or complexity of gastrointestinal microbial flora [3]. As far as inadequacy of methods is concerned, bacteria implicated may be in viable but not cultivable form as happens in most of the chronic infections. Further, less sensitive conventional methods of detection may be the reason for the nondetection of specific pathogen/s in UC cases. However, in the recent past extremely sensitive molecular method, nested PCR in particular has been found to detect as low as 3 microbes per clinical specimen [13]. Nested PCR takes care of PCR inhibitors present in the biological samples. Therefore, we decided to see the presence of* Salmonella species* in antral biopsy and stool specimens collected from UC, IBS, colon cancer, and healthy control to explore the possibility of its association with UC in the Eastern part of Northern India.

## 2. Materials and Methods 

### 2.1. Study Population

A total of 404 samples having mean age 36.21 (±13.639) were taken among which 59 cases of ulcerative colitis, 28 of colon cancer, 127 of irritable bowel syndrome, and 190 cases of apparently healthy controls were included in the present study conducted from July 2009 to February 2015 from inpatients and outpatient department of S. S. Hospital, Banaras Hindu University, Varanasi. The diagnosis of the patients with UC and colon cancer was done by lower gastrointestinal endoscopy, histopathology of rectal biopsy, and CT scan. Diagnosis of IBS was done by lower gastrointestinal endoscopy and Rome III classification/criteria. All the patients included were having clinical history of the disease. Patients having comorbid systemic illness, HIV, and mental and emotional disorders were excluded from the study.

### 2.2. Sample Collection and Processing

Rectal biopsies of the patients having positive endoscopic finding and stool samples from healthy control were taken after well informed written consent. Two to three rectal biopsies were taken from the same site of infection. About 5 mL of blood was collected by venipuncture aseptically in a sterile clot activator vial. Serum was separated and subjected for Widal and Indirect Haemagglutination Assay (IHA) and specimens were preserved at −80°C for further use.

### 2.3. Serological Studies

Widal test was performed by using standard protocol given by manufacturer's guideline (Span Diagnostics, India). The antibodies titre ≥1 : 160 against TO, TH, and AH antigen was considered significant in the present study. For the IHA test, antibodies against Vi antigen (ViAb) were measured following the method described by Barrett [14] and a titer of ≥160 was considered as significant to diagnose chronic typhoid carriers.

### 2.4. Molecular Studies

#### 2.4.1. Extraction of Genomic DNA from Clinical Specimens

Extraction of genomic DNA from rectal biopsies and stool samples was done using modified phenol-chloroform and proteinase-K method described by Sambrook and Russell and Van Zwet et al. [15, 16].

#### 2.4.2. Detection of Salmonella Typhi and Paratyphi A Targeting Specific Gene Sequences

In the PCR reaction, about 100 ng quantity of extracted genomic DNA from rectal biopsy and stool specimens were subjected to specific gene sequences amplification of Typhi and Paratyphi A. The primers used in this study were designed by Song et al. [17] which was further modified by Frankel [18]. However, for* staA* gene of* S*. Typhi and stkG gene of *S*. Paratyphi A, primers used were taken from the previous studies [19, 20] (Table 1).

#### 2.4.3. PCR Assay

The reaction mixture for the first-round PCR contained 2.5 *μ*L of 10x PCR buffer (100 mM Tris-HCl, 1.5 mM MgCl_2_, 50 mM KCl) (Genei, India), 10 pmol of each primer (*staA* F1,* staA* R1 and ST1, ST2 for* S*. Typhi;* stkG* F1,* stkG* F2 for* S*. Paratyphi A) (SBS Genetech Co., Ltd., China), 2 *μ*L (2.5 mM each) of dNTPs mix (Genei, India), 0.33 *μ*L (1 unit) of Taq DNA polymerase (Genei, India), and 5 *μ*L of DNA template (100 ng), and final volume of 25 *μ*L was adjusted with HPLC-grade sterile water. The amplification reaction was performed in a thermal cycler (Biometra, Germany). The nested PCR master mix was the same as that of the first-round PCR, except that it contained 10 pmol of each of the primers* staA* F2,* staA* R2 (ST1 and ST2) for* S*. Typhi and* stkG *F2 and* stkG *R2 for* S*. Paratyphi A and 2 *μ*L of DNA template (1 : 5-diluted product of the primary cycle). Thermal cycling was carried out as described for first-round PCR, except that the annealing temperature was set to 65°C (63°C for ST3 and ST4) and 61°C, respectively (Table 1). The amplification was repeated 2-3 times to ensure that the amplification obtained with the primers is reproducible and consistent.

The amplified DNA fragments were resolved through electrophoresis in 1.5% agarose gel prepared in TBE buffer and visualized in a gel documentation system (Alfa Imager 2200, Alfa Innotech Corporation, USA).

#### 2.4.4. Statistical Analysis

Statistical analysis was done by using *Z*-test to analyze the level of significance between two proportions, that is, nested PCR and serological test in the diseases of ulcerative colitis, colon cancer, and irritable bowel syndrome and healthy controls.

## 3. Results and Discussion

**Figure 1:**
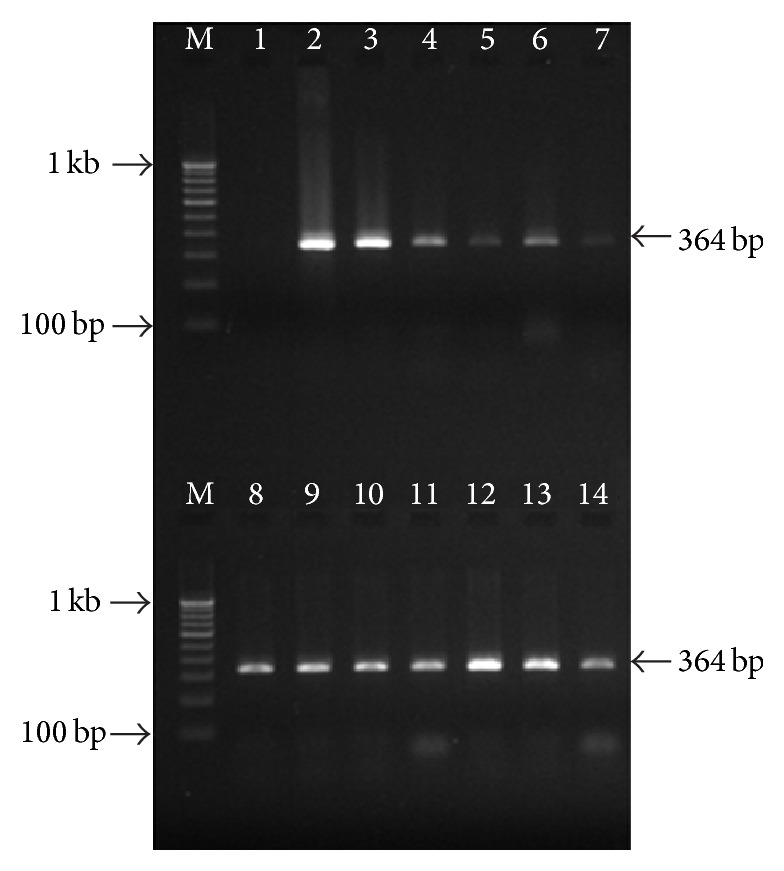
Gel picture showing the nested PCR amplification targeting flagellin (*fliC*) gene of* S*. Typhi in clinical specimens. Lane M, 100 bp DNA ladder; lane 1, negative control; lane 2, positive control (*S. *Typhi MTCC 3216); lanes 3–14, positive nested PCR amplification in different clinical samples.

**Figure 2:**
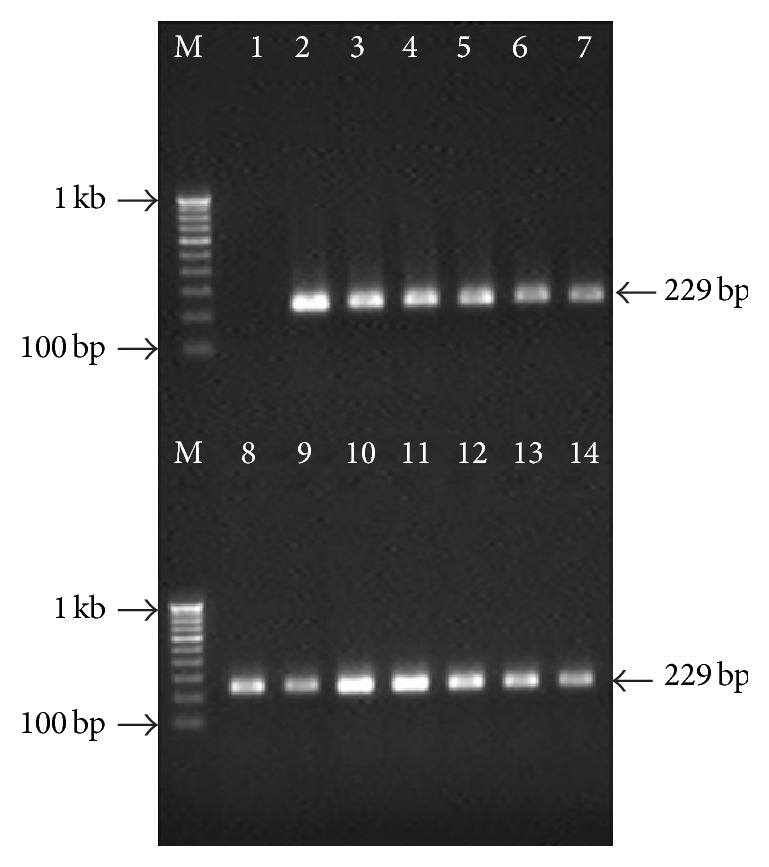
Gel picture showing the nested PCR amplification targeting putative fimbrial protein (*stkG*) gene of* S*. Paratyphi A in clinical specimens. Lane M, 100 bp DNA ladder; lane 1, negative control; lane 2, positive control (*S. *Paratyphi A ATCC9150); lanes 3–14, positive nested PCR amplification in clinical samples.

Ulcerative colitis (UC) patients were found to have highest PCR positivity (79.7%) for the* Salmonella enterica* serotypes (Typhi and Paratyphi A both) followed by IBS (66.1%), CC (53.6%), and HC (16.3%) in descending order. While the positivity rates for the* Salmonella* serotypes were statistically comparable, UC had significantly higher prevalence (*p* < 0.001) when comparing positivity rates in IBS, CC, and HC groups (Table 2, Figures 1 and 2). The positivity by PCR in healthy individuals shows 16.3% due to asymptomatic infection of the organism because PCR and serological based detection of* Salmonella* bacteria, the antibody titre, and positivity rate by molecular methods increase simultaneously in healthy individuals living in this endemic area.

Antibody titre (≥1 : 160) against Vi antigen usually showing chronic carrier state was lowest in UC (6.8%) compared to HC (12.1%) and this difference was statistically significant (*p* < 0.05) (Table 2). Antibody response at the titres (≥1 : 160) against somatic (TO), flagellar antigens of serotypes Typhi (TH) and Paratyphi A either in combination or alone was observed to be lowest again in UC patients (54.2%) in comparison with IBS (70.1%) and CC (71.4%). Healthy controls had the lowest percentage of individuals with positive Widal test (15.2%) (Table 3). On statistical analysis, it was observed that all the 3 diseases groups had significantly higher (*p* < 0.001) positivity rates as compared to healthy controls (HC). When antibody titre against TO was taken into account, 35.5% of patients with CC were observed with titre of ≥1 : 160 followed by UC (18.7%), HC (15.3%), and IBS (12.6%). On statistical analysis, the positivity percentage of CC was significantly higher than IBS (*p* < 0.001). Contrary to this the antibody titre at ≥1 : 160 against TH, IBS group had highest positivity rate (67.7%) followed by CC (57.0%), UC (39.0%), and HC (12.6%). UC patients were observed with significantly lower percentage of patients with significant titre against TH (*p* < 0.001) when compared with IBS. While UC and CC had comparable proportions of the patients with significant titre against TH, all the diseased groups showed significant titre (*p* < 0.001) (Table 2). The antibody titre against AH was observed at very low levels in all the 3 diseased groups and HC and these positivity percentages were comparable. The higher titers in diseased group indicate that these patients are more susceptible to either* Salmonella* infection or more responses to the* Salmonella* antigens resulting in autoimmune type of injury. In the previous report from our centre we have already established that PCR based detection of* Salmonella* bacteria and antibody responses including against ViAb go parallel in healthy individuals living in this endemic area [21].

The age-old definition of UC includes the phrase “presence of bloody diarrhea and mucous associated with negative stool culture for bacteria, ova, or parasites” with the diagnostic facilities available at that time. In the recent time with the advent of extremely sensitive and specific tools this definition may not be true. In the present study, efforts were made to look for* Salmonella enterica* serotypes Typhi and Paratyphi A (known causative agents of enteric fever) in patients with chronic gut diseases like UC, IBS, and CC, a malignant condition. By using nested PCR for detection of* S*. Typhi and Paratyphi A serotypes in stool samples, almost 80% of the UC patients were found to have the DNA of the bacterium which was significantly higher than the IBS and CC. It is well established that, due to lacunae in our isolation techniques and ignorance about growth triggering factors, the isolation rate from any clinical specimens including stool and colonic biopsies usually remains <40% [22, 23]. Such a high level of association with UC indicates two possibilities; that is, firstly* Salmonella *either is an aetiological agent or may be associated with this disease due to increased susceptibility to the infection due to loss of mucosal integrity. The possibility of* Salmonella* being one of the causative agents seems to be quite likely. This hypothesis is well supported by the observation that antibody titre against Vi antigen was lowest in UC patients (6.8%) suggesting active infection rather than chronic carrier state as Vi antibody response is usually more pronounced in chronic typhoid carriage. The possibility of the specific immunosuppression against* Salmonella* in UC patients cannot be ignored on the basis of this observation. The later speculation is further supported by quite low Widal positivity in UC cases (54.2%) as compared to IBS (70.1) and CC (71.4%). It is interesting to note that, despite the highest positivity rate for the presence of* Salmonella* DNA in UC, the antibody titre against somatic antigen (TO) of* Salmonella* was simply comparable to CC and IBS. Somatic antigen of the* Salmonella* is known to induce short lasting IgM response usually in acute infections. Following similar pattern, the antibody titre against flagellar antigen (TH) which is known to induce prolonged IgG response in UC patients was observed to be minimum (39.0%) amongst the diseased groups when compared with IBS (67.7%) and CC (57.0%). Therefore, the significantly higher positivity rate for* Salmonella* nucleic acid along with relatively poor immune response strongly suggests the aetiological role of the enteric pathogens in UC. These observations were supported by previous studies reporting the higher prevalence of enteric pathogens and UC in developing countries [6–11]. Further, there is report from North India showing association between poor hygiene and UC [24]. There are many reports implicating* Salmonella* and* Campylobacter enteritis* with increased short- and long-term risk for inflammatory bowel diseases including UC enteritis [9, 10]. There are few case reports published in 70s showing the isolation of* Salmonella* serotypes (Typhi, Typhimurium, and Agona) from patients who were initially admitted to infectious disease hospital during acute attacks of UC [11]. But the question that may be asked is why not is it merely an association rather than a causation which is quite likely in population with poor hygiene as is in the present study subjects? The point goes in favor of this speculation: why antibiotics alone are not giving the remission? It is anti-inflammatory drugs which include corticosteroids bringing remission. Therefore, UC may be an inflammatory bowel disease due to autoimmune aetiology. However, the autoimmunity might have been induced by some facultative/obligate intracellular microbial agent/s causing chronic infection and* Salmonella* spp. may be one such agent. We cannot deny the inadequacy of diagnostic methods and shooting in dark as far as UC is concerned. Nested PCR based technique is known for its sensitivity and specificity, though sensitivity also brings in the risk of PCR contamination. However, if we analyze the data regarding IBS, it may be appreciated that this entity is more likely due to immunological reaction rather than bacterial invasion itself as immune response follows closely with the rate of detection of* Salmonella* serotypes by PCR in this group. Chronic typhoid carriage is sometimes implicated in causation of CC apart from cancer of gallbladder and PCR based detection and immunological response might be the reflection of Typhoid carriage itself.

## 4. Conclusions

This is the preliminary study and further exploration is required. However, we hypothesize that direct invasion by some microbial agents is associated with hypersensitive state of immunological response in ulcerative colitis at least.

## Figures and Tables

**Table 1:** Primers used for amplification of *S*. Typhi and *S. * Paratyphi A serotypes in clinical specimens.

Primer sequence	Target gene	*Salmonella *serotype	Amplicon size (bp)	PCR condition (number of cycles)	References
ST1 5′-ACTGCTAAAACCACTACT-3′ ST2 5′-TTAACGCAGTAAAGAGAG-3′	*fliC*	Typhi	495 bp	94°C, 1 min; 52°C, 1 min; 72°C, 1 min (35)	Song et al. [17]
ST3 5′-AGATGGTACTGGCGTTGCTC-3′ ST4 5′-TGGAGACTTCGGTCGCGTAG-3′			364 bp	94°C, 1 min; 63°C, 1 min; 72°C, 1 min (35)	Frankel [18]

*staA* F1: 5′-TGGTTACATGACCGGTAGTC-3′ *staA* R1: 5′-TAGCTGCCGCAATGGTTATG-3′	*staA*	Typhi	537 bp	94°C, 1 min; 57°C, 1 min; 72°C, 1 min (35)	Pratap et al. [19]
*staA* F2: 5′-CATCGGCACGAACGTAAGAC-3′ *sta*A R2: 5′-TCAAGCGACTGATGGTGACG-3′			377 bp	94°C, 1 min; 65°C, 1 min; 72°C, 1 min (35)	

*stkG* F1: 5′-CGTTTACTGAGGTCACAGGCATC-3′ *stkG* R1: 5′-CACATTGTTCTCGGAGACCCCA-3′	*stkG*	Paratyphi A	427 bp	94°C, 1 min; 52°C, 1 min; 72°C, 1 min (35)	Pratap et al. [20]
*stkG* F2: 5′-CAATGGCTTCTGGCGAACTGTC-3′ *stkG* R2: 5′-TGGAGAAAGATCAGACCACCGAG-3′			229 bp	94°C, 1 min; 61°C, 1 min; 72°C, 1 min (35)	

**(a):** 

	Number of study subjects	Nested PCR		Vi positivity		Widal titre					
Study groups		Positivity		(titre ≥ 1 : 160)		TO		TH		AH	
		No.	%	No.	%	No.	%	No.	%	No.	%
Ulcerative colitis (UC)	59	47	79.7	4	6.8	11	18.6	23	39.0	2	3.4
Irritable bowel syndrome (IBS)	127	84	66.1	14	11.0	12	12.6	86	67.7	2	1.6
Colon cancer (CC)	28	15	53.6	3	10.7	10	35.5	16	57.1	1	3.6
Apparently healthy controls (HC)	190	31	16.3	23	12.1	29	15.3	24	12.6	2	2.1

**(b):** 

Nested PCR				
UC	Vs	IBS	*p* = 0.060	*Z* = 1.88
UC	Vs	CC	*p* = 0.050	*Z* = 2.51
UC	Vs	HC	*p* < 0.001	*Z* = 9.16
IBS	Vs	CC	*p* = 0.210	*Z* = 1.25
IBS	Vs	HC	*p* < 0.001	*Z* = 9.04
CC	Vs	HC	*p* < 0.001	*Z* = 4.51

ViAb				
UC	Vs	IBS	*p* = 0.36	*Z* = 0.91
UC	Vs	CC	*p* = 0.53	*Z* = 0.63
UC	Vs	HC	*p* = 0.25	*Z* = 1.15
IBS	Vs	CC	*p* = 0.98	*Z* = 0.05
IBS	Vs	HC	*p* = 0.77	*Z* = 0.29
CC	Vs	HC	*p* = 0.83	*Z* = 0.21

Widal				

TO				
UC	Vs	IBS	*p* = 0.080	*Z* = 1.77
UC	Vs	CC	*p* = 0.080	*Z* = 1.73
UC	Vs	HC	*p* = 0.530	*Z* = 0.62
IBS	Vs	CC	*p* < 0.001	*Z* = 3.61
IBS	Vs	HC	*p* = 0.130	*Z* = 1.51
CC	Vs	HC	*p* < 0.010	*Z* = 2.63

TH				
UC	Vs	IBS	*p* < 0.001	*Z* = 3.70
UC	Vs	CC	*p* = 0.120	*Z* = 1.59
UC	Vs	HC	*p* < 0.001	*Z* = 4.52
IBS	Vs	CC	*p* = 0.280	*Z* = 1.07
IBS	Vs	HC	*p* < 0.001	*Z* = 10.1
CC	Vs	HC	*p* < 0.001	*Z* = 5.68

AH				
UC	Vs	IBS	*p* = 0.42	*Z* = 0.79
UC	Vs	CC	*p* = 0.92	*Z* = 0.04
UC	Vs	HC	*p* = 0.21	*Z* = 1.25
IBS	Vs	CC	*p* = 0.48	*Z* = 0.69
IBS	Vs	HC	*p* = 0.68	*Z* = 0.41
CC	Vs	HC	*p* = 0.28	*Z* = 1.07

**(a):** 

Study groups	Number of study subjects	Positive	
		Number	%
Ulcerative colitis (UC)	59	32	54.2
Irritable bowel syndrome (IBS)	127	89	70.1
Colon cancer (CC)	28	20	71.4
Apparently healthy controls (HC)	190	29	15.2

**(b):** 

Widal				
UC	Vs	IBS	*p* < 0.050	*Z* = 2.11
UC	Vs	CC	*p* = 0.210	*Z* = 1.53
UC	Vs	HC	*p* < 0.001	*Z* = 6.08
IBS	Vs	CC	*p* = 0.890	*Z* = 0.142
IBS	Vs	HC	*p* < 0.001	*Z* = 9.89
CC	Vs	HC	*p* < 0.001	*Z* = 6.65

## References

[1] Danese S., Fiocchi C. (2006). Etiopathogenesis of inflammatory bowel diseases. *World Journal of Gastroenterology*.

[2] Loftus E. V. (2004). Clinical epidemiology of inflammatory bowel disease: incidence, prevalence, and environmental influences. *Gastroenterology*.

[3] Campieri M., Gionchetti P. (2001). Bacteria as the cause of ulcerative colitis. *Gut*.

[4] Lakatos L., Lakatos P. L. (2006). Is the incidence and prevalence of inflammatory bowel diseases increasing in Eastern Europe?. *Postgraduate Medical Journal*.

[5] Adams S. M., Bornemann P. H. (2013). Ulcerative colitis. *American Family Physician*.

[6] Jess T., Simonsen J., Nielsen N. M. (2011). Enteric *Salmonella* or *Campylobacter* infections and the risk of inflammatory bowel disease. *Gut*.

[7] Chassaing B., Darfeuille-Michaud A. (2011). The commensal microbiota and enteropathogens in the pathogenesis of inflammatory bowel diseases. *Gastroenterology*.

[8] Ferreira R. B. R., Gill N., Willing B. P. (2011). The intestinal microbiota plays a role in Salmonella-induced colitis independent of pathogen colonization. *PLoS ONE*.

[9] Ternhag A., Törner A., Svensson Å., Ekdahl K., Giesecke J. (2008). Short- and long-term effects of bacterial gastrointestinal infections. *Emerging Infectious Diseases*.

[10] Gradel K. O., Nielsen H. L., Schønheyder H. C., Ejlertsen T., Kristensen B., Nielsen H. (2009). Increased short- and long term risk of inflammatory bowel disease after *Salmonella* or *Campylobacter* gastroenteritis. *Gastroenterology*.

[11] Dronfield M. W., Fletcher J., Langman M. J. S. (1974). Coincident *Salmonella* infections and ulcerative colitis: problems of recognition and management. *British Medical Journal*.

[12] Sood A., Amre D., Midha V. (2014). Low hygiene and exposure to infections may be associated with increased risk for ulcerative colitis in a North Indian population. *Annals of Gastroenterology*.

[13] Prakash P., Mishra O. P., Singh A. K., Gulati A. K., Nath G. (2005). Evaluation of nested PCR in diagnosis of typhoid fever. *Journal of Clinical Microbiology*.

[14] Barrett T. J. (1985). Improvement of the indirect hemagglutination assay for *Salmonella typhi* Vi antibodies by use of glutaraldehyde-fixed erythrocytes. *Journal of Clinical Microbiology*.

[15] Sambrook J., Russell D. W. (2001). *Molecular Cloning: A Laboratory Manual*.

[16] Van Zwet A. A., Thijs J. C., Kooistra-Smid A. M. D., Schirm J., Snijder J. A. M. (1994). Use of PCR with feces for detection of *Helicobacter pylori* infections in patients. *Journal of Clinical Microbiology*.

[17] Song J.-H., Cho H., Park M. Y., Na D. S., Moon H. B., Pai C. H. (1993). Detection of *Salmonella typhi* in the blood of patients with typhoid fever by polymerase chain reaction. *Journal of Clinical Microbiology*.

[18] Frankel G. (1994). Detection of *Salmonella typhi* by PCR. *Journal of Clinical Microbiology*.

[19] Pratap C. B., Kumar G., Patel S. K. (2013). Targeting of putative fimbrial gene for detection of *S*. typhi in typhoid fever and chronic typhoid carriers by nested PCR. *Journal of Infection in Developing Countries*.

[20] Pratap C. B., Kumar G., Patel S. K. (2014). Mix-infection of *S. Typhi* and ParaTyphi A in typhoid fever and chronic typhoid carriers: a nested PCR based study in North India. *Journal of Clinical and Diagnostic Research*.

[21] Nath G., Maurya P., Gulati A. K. (2010). Comparison of Vi serology and nested PCR in diagnosis of chronic typhoid carriers in two different study populations in typhoid endemic area of India. *Southeast Asian Journal of Tropical Medicine and Public Health*.

[22] Ueda Y., Suzuki N., Miyagi K. (1997). Studies on bacillary dysentery cases of overseas travellers—during 1979 to 1995. *Nippon Saikingaku Zasshi*.

[23] Barbut F., Beaugerie L., Delas N., Fossati-Marchal S., Aygalenq P., Petit J.-C. (1999). Comparative value of colonic biopsy and intraluminal fluid culture for diagnosis of bacterial acute colitis in immunocompetent patients. *Clinical Infectious Diseases*.

[24] Khosla S. N., Girdhar N. K., Lal S., Mishra D. S. (1986). Epidemiology of ulcerative colitis in hospital and select general population of northern India. *The Journal of the Association of Physicians of India*.

